# Active electroceutical treatment of *Pseudomonas aeruginosa* infected murine wounds

**DOI:** 10.1371/journal.pone.0331785

**Published:** 2025-09-22

**Authors:** Colin R. Mack, Traci A. Wilgus, Sheri Dellos-Nolan, Tony DiCesare, Daria Bentley, Fraser J. Daniel, Vish V. Subramaniam, Sahil Mahajan, Paul Stoodley, Daniel J. Wozniak, Shaurya Prakash

**Affiliations:** 1 Department of Biomedical Engineering, The Ohio State University, Columbus, Ohio, United States of America; 2 Department of Pathology, The Ohio State University, Columbus, Ohio, United States of America; 3 Department of Microbial Infection and Immunity, The Ohio State University, Columbus, Ohio, United States of America; 4 Department of Microbiology, The Ohio State University, Columbus, Ohio, United States of America; 5 Department of Mechanical and Aerospace Engineering, The Ohio State University, Columbus, Ohio, United States of America; 6 Department of Orthopedics, The Ohio State University, Columbus, Ohio, United States of America; Pennsylvania State University Hershey Medical Center, UNITED STATES OF AMERICA

## Abstract

Chronic, infected dermal wounds have a high burden of cost and remain a persistent hurdle within healthcare. Electroceutical dressings that deliver antimicrobial agents directly to the wound site have emerged as an alternative solution to current standards of care. Here, through a systematic evaluation in an infected murine wound model we report on an actively powered electroceutical dressing that generates hypochlorous acid *in situ* as an antimicrobial agent to clear infection. In a significant new discovery, we also report that the treatment of these infected wounds with the actively powered electroceutical promotes the closure of wounds better than other test conditions. In a 10-day murine study, mice were wounded, and the dermal injury was infected with *Pseudomonas aeruginosa*. Four treatment plans were administered to these wounds: no treatment, a commercially available electroceutical dressing, and our electroceutical dressing both powered and unpowered. The mice were subsequently sacrificed 8 days post infection. The powered electroceutical dressing demonstrated significantly reduced bacterial burden compared to untreated wounds. The powered electroceutical dressing also showed the highest wound closure with nearly 60% wound area reduction and approximately 36% percent re-epithelization of the wound bed in contrast to the no-treatment case. When compared against the other treatments, the powered electroceutical dressing demonstrated an improved capability in promoting wound area reduction while lowering infection burden.

## Introduction

Infected dermal injuries continue to be a major clinical challenge due to the complex interplay between various infectious microorganisms and the wound microenvironment [1]. Wounds are characterized as acute or chronic, depending on the length of time without resolution [2]. In humans, acute wounds can resolve in 0–4 weeks post-treatment, but chronic wounds are injuries that do not heal and remain open from 4 weeks to three months of treatment [2–4]. In the United States, approximately 2% of the population is affected by chronic dermal injuries, with the subsequent global wound care cost estimated at ~$19 billion by 2027 [5]. One characteristic trait of chronic wounds in contrast to acute wounds is difficult-to-treat infections due to biofilms [6]. Biofilms are bacteria that accumulate together surrounding themselves in an extracellular polymeric substances (EPS) that are self-produced [7]. In one study, 60% of chronic wounds were found to contain biofilms compared to only 6% in acute wounds [8]. Moreover, wound biofilms show recalcitrance towards standard antimicrobial treatments [6,9,10]. Other characteristics of chronic wounds are presence of high levels of inflammatory cells, changes in local metabolic activity, elevated protease activity, defective extracellular-matrix, and limited re-epithelialization [11–15]. Often, infections in chronic wounds tend to be polymicrobial [6,8,16]. Despite the many treatment options available like debridement, antibiotics (systemic and topical), negative pressure wound therapy, and use of antiseptic agents; the challenges associated with disinfecting and healing chronic wounds continue to be reported in both the scientific and clinical literature [4,15,17–21]. Therefore, clearance of infection in a dermal injury is essential to resolve wounds.

Despite the existence of a myriad of antimicrobial agents and treatment strategies, the persistence of wound infections has led to the development of a new class of wound dressings called electroceuticals [22,23]. These emerging dressings are either passive, exploiting open circuit potentials and electric fields to generate reactive oxygen species (ROS) such as hydrogen peroxide through well-established redox chemistry supporting migration of keratinocytes [24], or with active electrical current flow to drive electrochemistry while providing additional electrical stimulation the dermal wound site [25,26]. The mechanisms and role of electrical stimulation of wounds is an active research area with many unanswered questions.

Passive electroceuticals, sold under brand names of Arthrex® and Procellera®, are commercially available with US Food and Drug Administration (US FDA) approval for disinfecting shallow (< 0.25 mm deep) wounds. These passive electroceuticals function through the generation of hydrogen peroxide as a disinfectant due to the redox chemistry arising from the use of dissimilar metals [24]. Passive dressings lack tunability or control over the generated redox potentials and therefore show poor regulation of any subsequent electrochemistry as the primary electrochemical control arises from equilibrium Nernst potentials [27]. On the other hand, active electroceuticals use a power source like a battery to generate flow of direct current (DC) which, in our case, produces the antimicrobial agent hypochlorous acid (HOCl) to inhibit bacterial growth. Our active electroceuticals have previously been demonstrated for use both *in vitro* and *in vivo* [22,23,28–31] with the efficacy of *in situ* generation of HOCl as an antimicrobial agent demonstrated previously. The use of active electrical power sources allows for significantly greater tunability in controlling not only local electrochemistry but also delivering defined electrical stimulation to the wound site. As with all engineered systems, the trade-off for controlled electrical stimulation is that active electroceutical dressings are more complex to manufacture than passive electroceuticals.

We have previously reported on an active electroceutical device (OSU electroceutical dressing or OSU ED), which comprises a screen-printed, battery-powered electrode on silk to achieve tunable application of electric current [28]. The OSU ED exploits fundamental principles of electrochemistry, generating HOCl a known antimicrobial agent as the primary disinfection agent, *in situ* [32,33]. The main advantage of the active OSU ED is its tunability, thereby permitting control of the electrochemistry to generate HOCl by controlling the electrode potentials. By regulating potentials, electric current flow is also controlled and therefore the OSU ED allows regulation of “dosing” for the antimicrobial agents at the wound site. Moreover, a clinical case study has recently demonstrated the use of the OSU ED to successfully resolve chronic wounds in a dog and a cat [22]. It is noteworthy that while literature is rapidly growing on use of various electroceuticals there are currently no standards available to compare their operation. Moreover, there remains a poor understanding of methods of action of electroceuticals towards wound healing in the presence of infection.

The purpose of this work is to provide the first systematic comparison of various electroceutical treatments with similar electrical stimulation. Consequently, we report *in vivo* results for treatment of infected wounds with the OSU ED in an established mouse model with an excisional wound infected by *Pseudomonas aeruginosa* (PA). We also describe engineering advances for miniaturizing the OSU ED for application to a wound on a mouse. Finally, we report in detail the dual action of the OSU ED in infection treatment and simultaneously producing healing of the infected wounds.

## Results

A total of four treatment groups were studied, each with n = 6 mice split between two cages. The treatment groups included battery-powered OSU EDs (OSU ED-P), OSU EDs with no power source (OSU ED-UnP), a commercially available passive electroceutical dressing Procellera™ (CED), and Tegaderm™ alone (No Treatment). The OSU ED-UnP was used as a control to demonstrate the actively powered OSU ED provides clear advantages in wound treatment. The CED acts as a comparable, yet passive, electroceutical dressing, and the no treatment group acts as a negative control. The Materials and Methods section provides complete detail for the study timeline and the treatment details. All the reported values for infection mitigation and wound closure are from the final day of the study, day 8. Two mice in the OSU ED-UnP treatment group and one mouse in the OSU ED-P treatment group died prior to the conclusion of the study with all mice mortality occurring within 48-hours post-infection, and prior to the first dressing change. Since the mice deaths occurred early during the study it suggests that the OSU EDs had minimal impact. Noting the past observations from other studies with mouse mortality, we believe that the mice deaths are random as is commonly observed with this model. For example, some mortality is not uncommon in wound infection studies [34–39]. Past studies by other researchers have also shown mortality in mice due to bacterial infection is not uncommon with survival rates as low as 30% [40] with implication of *Pseudomonas aeruginosa* being more lethal than *Staphylococcus aureus* [34,36]. Lastly, as also shown in past work, the change in number of mice with electroceutical treatment conditions is not uncommon over the duration of the study and has also been reported for other electroceuticals [35]. It is worth noting that the use of the OSU ED-P has been demonstrated for safe use with humans [25] and also disinfection and healing of chronic wounds in both a dog and a cat [22] with no significant adverse events reported previously.

### Wound closure evaluation

**Fig 1:**
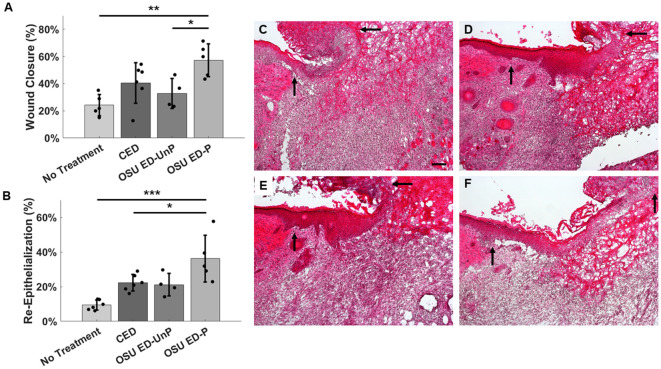
Effect of treatment on wound repair and regeneration. **(A)** Percent closure of the wound eight days post infection. Calculations were completed using wound area. Data expressed as mean ± SD; one-way ANOVA, *p* = 0.004. **(B)** Percent re-epithelialization of the wound eight days post infection. Data expressed as mean ± SD; one-way ANOVA, *p* < 0.001. For both **A** and **B**, dots in each bar indicate values for individual mice. **(C-F)** H&E-stained cross-sections of wound tissue. Arrows mark the wound edge from left to right for each image with **(C)** No Treatment, **(D)** CED, **(E)** OSU ED-UnP, and **(F)** OSU ED-P. Scale bar = 100 μm. *p < 0.05; **p < 0.01; ***p < 0.001.

As an evaluation of each treatment’s effect on wound closure and progression to resolution; percent wound area closure, percent wound re-epithelialization, and blood vessel density on day 8 was analyzed and compared across treatment groups. Fig 1A and Table 1 shows the percent wound area closure 8 days post infection. Of the four treatment groups, OSU ED-P demonstrated the greatest percent wound area reduction, determined by measurements taken from wound images, closing 57.2 ± 12.1% over the treatment sequence. The OSU ED-P significantly reduced *(p* = 0.003 and *p* = 0.048 respectively) the wound area compared with no treatment (24.2 ± 7.9% reduction) and OSU ED-UnP (32.8 ± 10.9% reduction). The CED (40.5 ± 15.0% reduction), which is only approved by the FDA for disinfection, showed no statistically significant difference in wound area reduction with respect to the no-treatment condition.

**Table 1:** Summary of the wound closure determined by reduction in wound area (%) and the percent re-epithelialization (%) of the wound bed for the mice across all treatment groups over the treatment duration. All values are expressed as mean ± SD.

	No treatment	CED	OSU ED-UnP	OSU ED-P
Wound closure (%)	24.2 ± 7.9	40.5 ± 15.0	32.8 ± 10.9	57.2 ± 12.1
Re-epithelialization (%)	9.4 ± 2.9	22.2 ± 4.8	21.2 ± 6.5	36.2 ± 13.4

The percent re-epithelialization of the wound was also compared across treatment groups (Fig 1B and Table 1). H&E-stained cross-sections of the wound tissue were used to determine the percentage of the wound bed (marked by black arrows) covered by the neoepidermis as shown in Fig 1C-1F. Of the four treatment groups, only OSU ED-P with an average percent re-epithelization of 36.2 ± 13.4% had a statistically significant greater percent re-epithelization (*p* < 0.001 and *p* = 0.048 res*p*ectively) than the mice that received no treatment (9.4 ± 2.9%) and those treated with the CED (22.2 ± 4.8%). The CED treated mice did not show a statistically significant increase in wound re-epithelialization compared to the no-treatment condition. Moreover, the OSU ED-UnP with a percent re-epithelialization of 21.2 ± 6.5%, was also not significantly different than the no treatment group. Blood vessel density was calculated using PECAM-1 stained cryosections of harvested tissue. Analysis of the blood vessel density across groups in the dermal tissue at the wound edge revealed a significant difference (*p* = 0.003) between the CED with a blood vessel density of 18.3 ± 5.1% and the OSU ED-P with a blood vessel density of 12.1 ± 1.8% (S1A Fig). Blood vessel density at the center of the wound, S1B Fig, showed no significant difference between treatment grou*p*s.

### Clearance *of* PA-Xen41 infection

**Fig 2:**
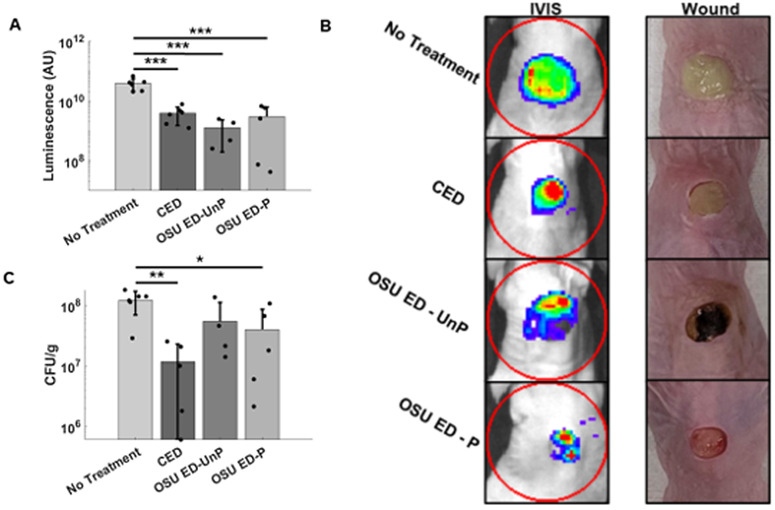
IVIS imaging and endpoint CFU show clearance of PAO1-Xen41 infection. **(A)** IVIS luminescence quantification (AU) for mice 8 days post infection showing the relative luminescence of the PA-Xen41. Data expressed as mean ± SD; one-way ANOVA, *p* < 0.001. **(B)** IVIS images from representative infected wounds in mice for each group. IVIS images of the wound eight days post-infection showing the bioluminescence of the PA-Xen41. In IVIS images, red indicates high bacterial luminescence and blue indicates lower bacterial luminescence. Accompanying wound images show a yellow hue in the no treatment wound group due to significant bacterial load. The OSU ED-UnP has a dark residue in the wound left behind from the Ag/AgCl ink. **(C)** Bacterial burden (CFU/g) of the wound eight days post-infection taken from the excised tissue of sacrificed mice. Data expressed as mean ± SD; one-way ANOVA, *p* = 0.006. *p < 0.05; **p < 0.01, ***p < 0.001.

The generation and delivery of antimicrobial agents is important for treating infected wounds. The OSU ED clearly demonstrates the ability to clear infection in an *in vivo* murine model. Fig 2A shows the clearance of the bacteria visually demonstrated through the luminescence of the PAO1-Xen41 on day 8 post infection. All three treatments (CED, OSU ED-UnP, OSU ED-P) were found to lower (*p* < 0.001 for all respectively) the luminescence (AU) when compared to the no treatment condition. The IVIS images in Fig 2B show a representative set of images explicitly demonstrating the decrease in the total bioluminescence signal. From the collected tissue samples, the bacterial load was quantified by CFU counts and compared across treatment groups for all mice on day 8 post infection (Fig 2C). A statistically significant lower average CFU count was found for both OSU ED-P (*p* = 0.050) and CED (*p* = 0.005) when compared to the mice that received no treatment. The OSU ED-UnP saw no significant decrease in CFU/g when compared to the mice that received no treatment.

### Local host immune response *to* infection and treatment

**Fig 3:**
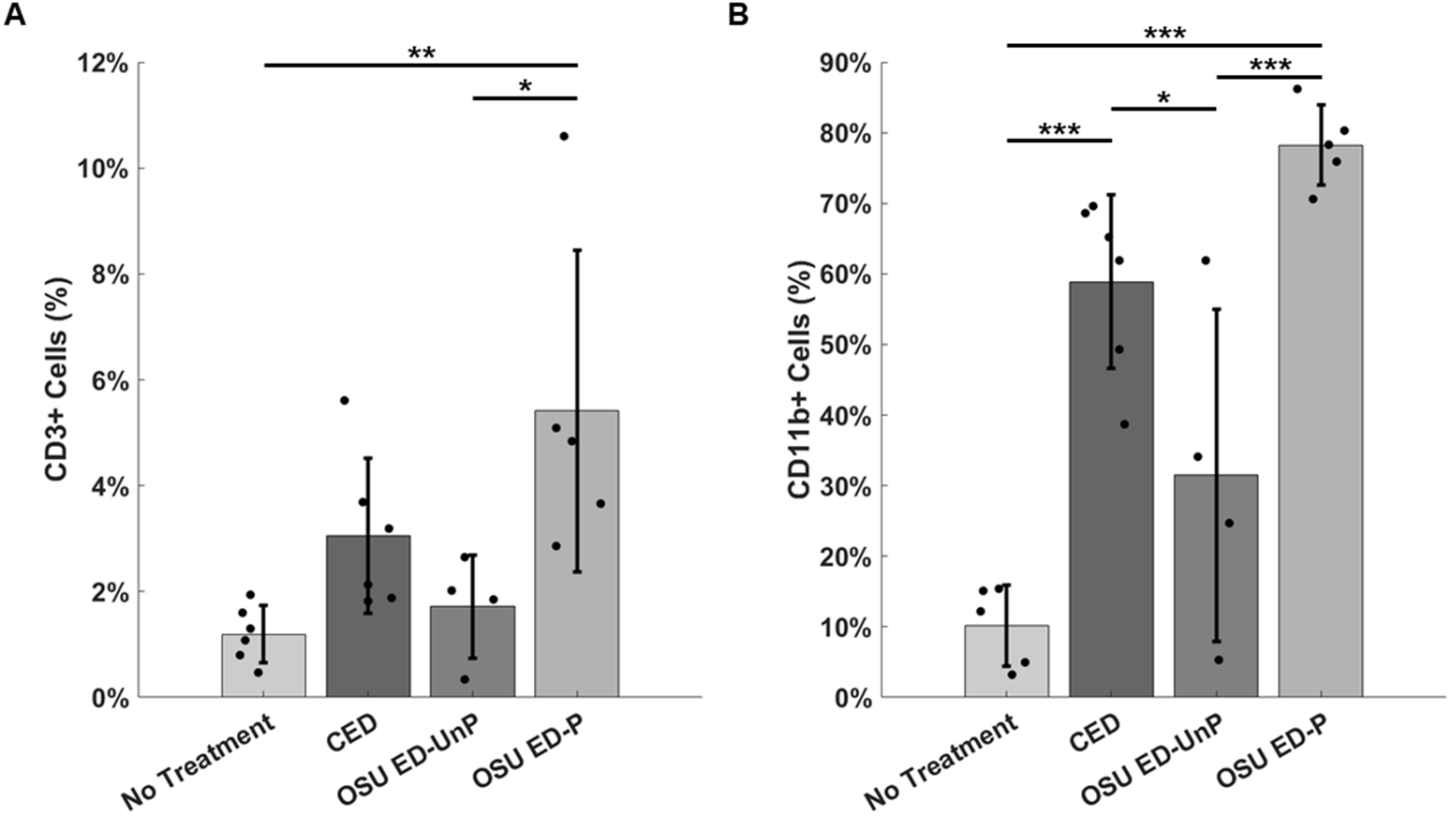
Local immune response of the mice. **(A)** Percent count of CD3 + cells counted by flow cytometry of excised wound tissue. Data expressed as mean ± SD; one-way ANOVA, **p* *= 0.006. **(B)** Percent count of CD11b+ cells counted during flowcytometry of excised skin. Data expressed as mean ± SD; one-way ANOVA *P* < 0.001. *p < 0.05; **p < 0.01, ***p < 0.001.

The local host immune response to infection and treatment was quantified through both flow cytometry and immunohistology. CD3 is a pan-T-cell marker expressed by and commonly used to detect T cells [41]. As a marker for T cells, CD3 + cells can be used as a measure of the host’s adaptive immune response. Therefore, the number of CD3 + cells were quantified in excised tissue through flow cytometry and compared across treatment groups (Fig 3A). OSU ED-P treated wounds had significantly greater CD3 + cells present than the no treatment group (*p* = 0.006) and the OSU ED-UnP (*p* = 0.035). The CED did not show a statistically significant response compared to either the no treatment or the OSU ED-UnP control groups. Similarly, CD11b+ cells were also evaluated as a general marker for the immune response in the murine infection model (Fig 3B). When compared to the no treatment control group, a significantly higher amount of CD11b+ cells were found in the mice treated with the CED and OSU ED-P (*p* < 0.001). Additionally, when compared to the OSU ED-UnP, a significantly higher amount of CD11b+ cells were found in the mice treated with the CED and OSU ED-P (*p* = 0.029 and *p* < 0.001 respectively). S2 Fig shows the representative density dot plots to determine the percentage of CD3+ or CD11b+ cells. When the macrophage density was compared across treatment groups by immunohistochemistry (S1C Fig), the CED and OSU ED-UnP both had statistically higher macrophage densities (32.3 ± 2.6% and 36.5 ± 4.1% respectively) in dermal tissue at the wound edge compared to the untreated group (25.4 ± 2.7%). The OSU ED-P was found to have significantly lower macrophage density of 29.8 ± 1.3% when compared to the OSU ED-UnP.

## Discussion

The objective of this 8-day study was to demonstrate proof-of-concept of antimicrobial action of the OSU ED and compare to other existing treatment controls with and without electrical stimuli. Wound healing in addition to disinfection was also observed. Disinfection was quantified using CFU/g as a metric compared to the untreated case. Past work with electroceutical dressings developed in our laboratories at The Ohio State University used a 6 V battery with a 10 kΩ ballast resulting in a maximum current flow of 600 µA for the treatment of chronic wounds on a cat and a dog [22]. For smaller animals (i.e., mice) used in the experiments here, the weight burden of the dressings had to be reduced. In our pilot studies with 2–4 mice, it was noted that this higher maximum current level or dose led to discomfort in mice as verified by an independent veterinarian. Notably, in another previous study by our team evaluating the safety of these dressings on human patients, the 6V, 10 kΩ ballast for limiting current was noted to be safe for human use [25]. However, in the human safety study, when the ballast resistance was reduced to 1 kΩ, one human subject reported an adverse event to wound treatment. Clearly, different wounds require distinct current and voltage settings for the electroceuticals. Therefore, the ability to control the applied potential and subsequent current flow are important engineering parameters. Hence, for this work, a lower voltage of 1.5 V was applied to dressing with a 15 kΩ ballast, reducing both the applied potential (by 4X) and current flow (by 6X) compared to our past work. It is noteworthy that under these engineered (and controllable) current and voltage conditions, the OSU ED-P is similar in voltage generated by the CED [42,43] and lower than the 3V reported by Fleming *et a*l. in their e-bandage [23]. Therefore, the OSU ED-P is tunable in “electrical dosing” and presents advances over other electroceutical dressings [26]. However, as described in the methods section, there are no standard metrics to apply or evaluate either the direct electrical or subsequent electrochemical dosing in wounds.

Of significance, the wound area reduction of the mice treated with OSU ED-P was noticeably greater than without treatment (Fig 1A). In contrast, CED showed no significant impact on wound healing. It is worth noting that the CED is a passive dressing, with no active current flow. The electric potential generated by the CED is a redox potential due to the silver (Ag) and zinc (Zn) metals in contact with the wound fluid. Moreover, the respective Ag and Zn patterns on the CED are electrically discontinuous. The Ag/Zn redox chemistry to generate an open circuit potential is well known, with Ag/Zn batteries used in the past to power satellites, deep water carriers, and medical devices [44]. The OSU ED-UnP showed no statistically significant wound closure compared to the no treatment condition. When measured, the OSU ED-UnP shows an open circuit potential of ~85 mV in 1X PBS which is comparable to a single Ag/Zn electrode pair of approximately 1 mm size exposed to PBS [24,45].

Furthermore, as seen with the results for percent re-epithelization of the wound bed (Fig 1B), the OSU ED-P performed significantly better than the CED. Similarly, the OSU ED-P also showed increased wound area reduction when compared to the OSU ED-UnP treated mice suggesting that active current flow limited to 100 μA along with 1.5V potential contributes positively to wound closure in the murine wounds evaluated here. In a recent advance, a wireless electroceutical by Jiang et al. [35] operating at a resonant frequency of 13.56 MHz with an antenna loading voltage of up to 6 V and current flow of 400 μA (compared to 100 μA here) has also demonstrated 25% increase in healing rate when compared to the untreated mice. The blood vessel density in the dermis at the wound edge for the OSU ED-P was lower than the CED as shown in S1A Fig. The CED blood vessel density at the wound edge was found to be not statistically significant between the untreated case and the other three treatment cases. The observations here suggest a nuanced outcome from the OSU ED-P treatment with the OSU ED-P treated wounds progressing further towards resolution in the same time-duration of treatment with the high-density capillaries that form in the earlier stages of wound healing, when angiogenesis occurs, and being pruned with continued wound closure [46]. During treatment with OSU ED wound discoloration from the Ag/AgCl ink can occur and has been observed in previous applications evaluating the OSU ED for use in humans [25] and in treatment of infected dog wounds [22]. The discoloration has not been shown to cause any adverse events nor impact histology measurements. Disinfection with electroceutical treatments has been reported previously [26,30,31,35,47]. Similarly, we have also previously reported that the OSU ED-P demonstrated a capability of mitigating bacterial infection both *in vitro* and *in vivo* [22,28,33] primarily through the production of HOCl. Past literature shows that the CED produces H_2_O_2_ for disinfection through well-known redox chemistry [24]. Other electroceuticals, such as the e-bandage and the wireless dressings have also shown disinfection, by generating electrochemical by-products that are broadly classified as reactive oxygen species (ROS) and are effective disinfectants for bacterial biofilms [35,48]. By contrast, the OSU ED-UnP did not demonstrate a significant drop in CFU/g when compared to the mice that received no treatment showing that the silver-electrodes by themselves did not contribute to bacterial eradication despite the presence of a low open circuit potential, and associated electric field, consistent with our past observations of *in vitro* electroceutical treatment of *P. aeruginosa* [33].

Given the finding of pro-healing benefits of the OSU ED-P (Fig 2), flow cytometry was performed on the cells derived from excised tissues. The local wound immune response is complex to the various electroceutical treatments. Mice treated with OSU ED-P demonstrated an increased number of CD3 + cells compared to the no treatment (control) condition suggesting the increased recruitment of naive T-cells and activated adaptive immune system. Mice treated with the OSU ED-P also showed a statistically significant increase in the CD11b+ cells compared to the no treatment case. Furthermore, the OSU ED-P treated mice also showed an increase in both CD3+ and CD11b+ cell counts compared to the OSU ED-UnP treated mice. On the other hand, the CED treated mice only showed an increase for the CD11b+ cell counts and not the CD3 + counts compared to the untreated case. CD11b is considered a pan-macrophage marker but can also be expressed by a variety of leukocytes including myeloid-lineage dendritic cells where its expression promotes pro-inflammatory responses [49,50]. Fig 3 shows that the percentage of both CD3+ and CD11b+ cells was significantly lower for the no-treatment condition compared to all other conditions. Therefore, it is reasonable to hypothesize that the electroceutical treatments provide a mechanism for local modulation of both inflammation and immune response in such manner as to mitigate infection and promote wound closure. However, a detailed mechanistic evaluation that includes a full panel of inflammatory cells and pro-inflammatory mediators is beyond the scope of this paper and is left for future work [35]. Wound healing requires changes in the wound milieu reflected in altered expression and concentrations of a variety of growth factors and cytokines [51]. These changes to the wound milieu are in-turn impacted by treatment methods. Future work using electroceuticals such as the OSU ED should further evaluate the comparative effect of treatment methods on specific proliferation markers and inflammatory indicators. In summary, this work has demonstrated the efficacy of the OSU ED-P in both bacterial clearance and wound closure in an infected murine wound model. Compared to past work, this study also demonstrated that the applied 1.5 V along with the current flow limited to 100 µA was impactful for both wound closure and bacterial infection mitigation in the murine wounds infected with PA. However, given the range of electrical stimulation parameters reported in literature, the exact mechanisms governing either wound healing or wound disinfection as function of “dosing” remain open questions. Furthermore, this work conducted a shorter duration study evaluating early-stages of wound healing and likely future work can evaluate longer treatment evaluation extending to 14 or 21-days as recently reported for another electroceutical [35]. In conclusion, the OSU ED-P treatment provides direct evidence for an actively powered electroceutical dressing with benefits for simultaneous wound healing and disinfection of infected murine wounds.

## Materials and methods

### Electroceutical dressing fabrication

**Fig 4:**
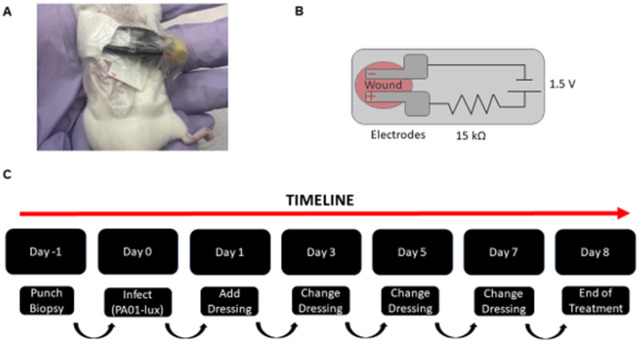
Device layout and experimental timeline of the evaluation of the OSU ED on infected wounds in mice. **(A)** OSU ED attached to the back of a mouse. The mouse shown in the image is from the pilot study and is shown for demonstration purposes with results here reported using hairless mice. **(B)** Schematic of the 1 cm x 2 cm OSU ED with a 1.5 V battery and a 15 kΩ ballast to limit current flow. **(C)** Schematic depicting timeline with the following events noted: punch biopsy to initiating wounding, infection of wound with PAO1-Xen41, dressing applications, and changes to dressings.

The OSU ED fabrication method and its safe use for humans has been previously reported [22,25,28]. Briefly, the OSU ED was screen-printed with medically compatible Ag/AgCl ink (Creative Materials #113−09) onto a habotai silk substrate (Fig 4A). In this work, to demonstrate the electrical tunability of the OSU ED, the dressing was powered by a 1.5 V battery (POWEROWL, LR41) with a 15 kΩ ballast resistor to limit the maximum current flow to 100 µA (Fig 4B) in contrast to past work which used a 6 V battery and a 10 kΩ current limiting resistor. Medical tape encapsulated all electrical components while providing an electrical and fluid isolation layer, leaving the printed electrodes and battery pack accessible for use. The use of silk as a flexible fabric substrate enabled the OSU EDs (1 cm x 2 cm) to be easily cut using common office scissors to fit the 8 mm wound on the mice. The size of these miniature OSU EDs is similar to those used previously for chronic wound treatment in a cat [22] with new advances to device engineering for the much smaller size of the mice. One key advance required for these mice wound studies was to reduce the total weight of the dressing with all supporting battery and electronic components to 1.2 g. This total weight change was necessitated due to a pilot study with 2 mice that showed discomfort as judged by an independent veterinarian when the total dressing weight was ~ 4 g with our previous dressings that were used for the treatment of the wound on the cat. With the reduced weight, the mice showed no restricted movement or visible evidence of discomfort. In total, two separate pilot studies were conducted to optimize the battery size and dressing tolerance with no adverse effects observed.

### Mouse wounding, infection, and treatment

All experiments were conducted in University Laboratory Animal Research facilities and treatments and procedures were performed in biosafety hoods. The protocols for wounding and infection followed methods reported previously [30,52,53] and were approved by the Ohio State University Institutional Animal Care and Use Committee (IACUC) under protocol #2017A00000033-R1. All methods were followed in accordance with the approved IACUC protocol #2017A00000033-R1 and are reported in accordance with ARRIVE guidelines. Briefly, 6-week-old female SKH1 hairless mice (Charles River Laboratories) were chosen for their immunocompetence, lack of pigment and hair benefiting the *in vivo* imaging system (IVIS), and for their use in wound healing models [52,54]. The SKH1 mice also allowed for better adherence of the dressings to the skin without animal discomfort. Since the mouse infection protocols have also been previously reported [52,53,55], only a brief description is provided here. Wounding was introduced on Day –1 (Fig 4C) with the mice anesthetized using isoflurane gas followed by use of an 8 mm biopsy punch to induce a full thickness dorsal wound. The wounds were then covered with Tegaderm™ and the mice were returned to their respective cages. Buprenorphine, an analgesic, was administered *via* sub-cutaneous injection. After a 24-hour recovery period (i.e., Day 0), the wounds were inoculated with 10^7^ cells of bioluminescent PAO1-Xen41 (*P. aeruginosa*) from an inoculum at a concentration of 10^8^ CFU/mL arising from an overnight bacterial culture [52,53]. The bacteria were allowed to grow in the wound bed over the next 24-hours to establish an infection as reported previously [52]. Steam sterilized, battery-powered OSU EDs (Fig 4A: OSU ED-P) were applied directly to the infected wounds 24 hours after inoculation, marked Day 1 in the experimental protocol (Fig 4C).

To provide a detailed comparison, additional mice in control groups were administered treatments as follows: the OSU electroceutical dressing with no power source (OSU ED-UnP), a commercially available passive electroceutical dressing Procellera™ (CED), or Tegaderm™ alone (no treatment). It is noteworthy that at present, no standard exists to compare electroceutical technologies for electrical parameters with respect to wound infection and healing. Therefore, the dressing operating conditions chosen here for the OSU ED-P uses a low voltage and current limited setting to compare systematically against the non-powered OSU ED (analogous to a passive dressing), and a commercial electroceutical dressing which generates only a potential difference and associated electric field without current flow to produce redox reactions at discontinuous metal patterns on a polyester substrate (CED).

Each group (No Treatment, CED, OSU ED-UnP, and OSU ED-P) had n = 6 mice divided between two cages (3 mice per cage). Not all mice survived the duration of the study with specific details discussed in the results section. Dressings were replaced every 48-hours, at which time wounds were photographed for wound size measurements and imaged with IVIS for measurements of bacterial bioluminescence and burden. On day 8, the mice were euthanized by CO_2_ inhalation. Post-sacrificing the mice, tissue from wounds were harvested and bisected. Half of the wound was embedded and frozen in TBS tissue-freezing medium (Triangle Biomedical Sciences, Durham, NC) for histological and immunohistochemical analysis. The other half was divided for residual bacterial colony forming unit (CFU) enumeration and flow cytometry analysis.

### Wound area calculation

The wound area was calculated from the wound images by using the methodology previously reported [22]. Briefly, for each wound a digital photographic image was recorded through a Samsung Galaxy Phone camera mounted on a tripod. Each digitized image was subsequently loaded into MATLAB® and processed to obtain the visible wound area. Using the Image Segmenter within MATLAB®, the area of the wound was obtained as pixels/mm [2] by using a standard of known size in each image as a physical reference. Further, within the Image Segmenter, a segmentation was created by hand-drawing a region of interest (ROI) that captures the visible boundaries of the wound. This was done in three replicates to minimize human error. The segmentations were then averaged and divided by the number of pixels/mm [2] to determine the wound area in mm [2]. This wound area along with the standard deviation of the segments were recorded. The wound area reduction was determined by subtracting the wound area Day 1 post infection from the wound area for the day of interest and dividing by the wound area Day 1 post infection and reported as a percent change.

### Histological and immunohistochemical analysis

10-μm cryosections from TBS-embedded wounds were prepared for histology and immunohistochemistry. The percent re-epithelialization was calculated as described previously [56,57] after using image analysis software to measure the total width of the wound bed and the distance covered by the neoepidermis. Hematoxylin and eosin (H&E)-stained wound sections were used to analyze wound re-epithelialization.

PECAM-1 (CD-31 marker) stained cryosections were prepared and used to quantify the blood vessel density as also described previously [56,58]. CD-31 is a recognized marker for endothelial cells and is considered a specific and sensitive marker for vascular differentiation, but is also expressed by platelets, macrophages, granulocytes, lymphocytes (T cells, B cells, and NK cells) [59]. Cryosections were also stained with F4/80 primary antibodies as also described previously [60]. F4/80 is a major macrophage marker, though it is also expressed by other immune cells [61]. Cell density (cells/mm [2]) was determined by counting the number of cells in a digital image and dividing by the area in which the cells were counted.

### Flow cytometry

Excised wound tissue was cut into small pieces with forceps and scissors. Tissue samples were then digested in DMEM containing 0.25 mg/mL Liberase™ TM (Thermolysin Medium) Research Grade (Roche Diagnostics) and DNase 1 (Millipore Sigma) for 2 h at 37^°^C with constant mixing. The digested cell suspension was filtered through 100 μm cell strainer. The red blood cells were lysed using the ACK lysis buffer for 5 mins. The cells were washed and finally resuspended in FACS buffer (PBS containing 1% BSA and 2 mM EDTA). For flow cytometry staining, cells were kept at 4^°^C and blocked with fluorochrome-conjugated anti-CD16/32 antibodies before being stained with primary antibodies for 30 mins. Cells were washed and fixed with BD Cytofix and acquired using BD LSRFortessa.

### Statistics

All statistical analysis was performed in SPSS Statistics (Armonk, NY, USA). For multiple comparisons between treatment groups for all mice, Analysis of Variance (ANOVA) was performed with Bonferroni post-hoc correction. Statistical significance was determined to a *p* < 0.05. Error bars reported for all data are plus and minus one standard deviation from the mean with values reported in the results represented as mean ± SD (standard deviation). Outliers for all data were determined using Grubbs’ test (*α* = 0.05) through GraphPad.

## Supporting information

S1 Fig Immunohistochemistry. Wound blood vessel density was determined by staining for PECAM and macrophage density was determined by immunohistochemical staining for F4/80. **(A)** Blood vessel density (%) of the re-epithelialized tissue adjacent to the center of the wound. Data expressed as mean ± SD; one-way ANOVA p = 0.011. **(B)** Blood vessel density (%) of the center of the wound absent of epithelial tissue. Data expressed as mean ± SD; one-way ANOVA p = 0.544. **(C)** Macrophage density (%) of the re-epithelialized tissue adjacent to the center of the wound. Data expressed as mean ± SD; one-way ANOVA p < 0.001. **(D)** Macrophage density (%) of the center of the wound absent of epithelial tissue. Data expressed as mean ± SD; one-way ANOVA p = 0.815. *p < 0.05; ** p < 0.01; *** p < 0.001.
(TIF)

S2 Fig Flow cytometry. Representative images of the density dot plots used in the flow cytometry analysis for **(A)** CD3+ and **(B)** CD11b+ cells.
(TIF)
