# Supplementary figures and images for: Active electroceutical treatment of Pseudomonas aeruginosa infected murine wounds
Source: PLoS One. 2025 Sep 22;20(9):e0331785. doi: 10.1371/journal.pone.0331785 (PMC12453229; doi:10.1371/journal.pone.0331785)

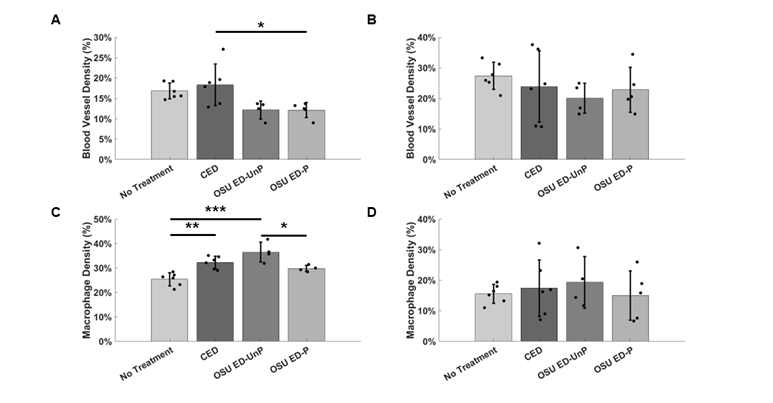

Supplement: S1 Fig — Wound blood vessel density was determined by staining for PECAM and macrophage density was determined by immunohistochemical staining for F4/80. (A) Blood vessel density (%) of the re-epithelialized tissue adjacent to the center of the wound. Data expressed as mean ± SD; one-way ANOVA p = 0.011. (B) Blood vessel density (%) of the center of the wound absent of epithelial tissue. Data expressed as mean ± SD; one-way ANOVA p = 0.544. (C) Macrophage density (%) of the re-epithelialized tissue adjacent to the center of the wound. Data expressed as mean ± SD; one-way ANOVA p < 0.001. (D) Macrophage density (%) of the center of the wound absent of epithelial tissue. Data expressed as mean ± SD; one-way ANOVA p = 0.815. *p < 0.05; ** p < 0.01; *** p < 0.001. (TIF) [file pone.0331785.s001.tif]

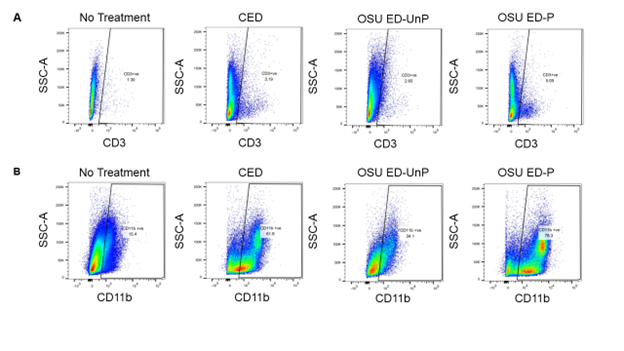

Supplement: S2 Fig — Representative images of the density dot plots used in the flow cytometry analysis for (A) CD3+ and (B) CD11b+ cells. (TIF) [file pone.0331785.s002.tif]
